# Chronic plantar heel pain modifies associations of ankle plantarflexor strength and body mass index with calcaneal bone density and microarchitecture

**DOI:** 10.1371/journal.pone.0260925

**Published:** 2021-12-09

**Authors:** Jason Andrew Rogers, Graeme Jones, Jill Cook, Kathryn Squibb, Karen Wills, Aroub Lahham, Tania Winzenberg

**Affiliations:** 1 Menzies Institute for Medical Research, University of Tasmania, Hobart, Tasmania, Australia; 2 La Trobe Sport and Exercise Medicine Centre, La Trobe University, Melbourne, Victoria, Australia; 3 Department of Health, Tasmanian Health Service, Scottsdale, Tasmania, Australia; 4 Department of Allergy, Clinical Immunology and Respiratory Medicine, Monash University, Melbourne, Victoria, Australia; 5 Faculty of Health, University of Tasmania, Hobart, Tasmania, Australia; University of Life Sciences in Lublin, POLAND

## Abstract

Chronic plantar heel pain (CPHP) is associated with calcaneal bone spurs, but its associations with other calcaneal bone features are unknown. This study therefore aimed to determine associations between having CPHP and bone density and microarchitecture of the calcaneus. We assessed 220 participants with CPHP and 100 age- and sex-matched population-based controls. Trabecular bone density, thickness, separation and number, BV/TV, and cortical density, thickness and area were measured using a Scanco Xtreme1 HR-pQCT scanner at a plantar and mid-calcaneal site. Clinical, physical activity and disease history data were also collected. Associations with bone outcomes were assessed using multivariable linear regression adjusting for age, sex, physical activity, BMI and ankle plantarflexor strength. We assessed for potential effect modification of CPHP on these covariates using interaction terms. There were univariable associations at the plantar calcaneus where higher trabecular bone density, BV/TV and thickness and lower trabecular separation were associated with CPHP. In multivariable models, having CPHP was not independently associated with any bone outcome, but modified associations of BMI and ankle plantarflexor strength with mid-calcaneal and plantar bone outcomes respectively. Beneficial associations of BMI with mid-calcaneal trabecular density (BMI-case interaction standardised X/unstandardised Y beta -10.8(mgHA/cm^3^) (se 4.6), thickness -0.002(mm) (se 0.001) and BV/TV -0.009(%) (se 0.004) were reduced in people with CPHP. Beneficial associations of ankle plantarflexor strength with plantar trabecular density (ankle plantarflexor strength -case interaction -11.9(mgHA/cm^3^) (se 4.4)), thickness -0.003(mm) (se 0.001), separation -0.003(mm) (se 0.001) and BV/TV -0.010(%) (se 0.004) were also reduced. CPHP may have consequences for calcaneal bone density and microarchitecture by modifying associations of BMI and ankle plantarflexor strength with calcaneal bone outcomes. The reasons for these case-control differences are uncertain but could include a bone response to entheseal stress, altered loading habits and/or pain mechanisms. Confirmation with longitudinal study is required.

## Introduction

Chronic plantar heel pain (CPHP) is a clinical condition that causes pain on the underside of the heel that is aggravated by weightbearing activity [1]. It is the most common reason why individuals with musculoskeletal foot pain consult a foot health practitioner [1], and is associated with significant foot-related disability and impaired quality of life (QOL) [2].

The plantar calcaneus is an important weightbearing bone that receives the proximal attachment of the plantar fascia. Degeneration of the plantar fascia at its enthesis [3], plantar fascial thickening [4] and plantar enthesophytes [4] are common findings in CPHP and are thought to be important in its aetiology. The consequences of these findings for calcaneal bone density and structure are currently unknown. As the plantar fascia transmits large forces through the plantar calcaneus [5], alteration in its structure or function in CPHP has the potential to influence calcaneal bone. There are also differences between cases and controls for potential bone modifying factors such as BMI [6] and ankle plantarflexor strength [6], yet nothing is known about how these factors influence calcaneal bone structure in CPHP.

The recent application of bone imaging tools such as high-resolution peripheral quantitative computed tomography (HR-pQCT) to the foot [7,8] means it is now possible to provide a detailed, in-vivo examination of bone in CPHP. As a highly trabecular bone [9] subject to large active and weightbearing forces, the calcaneus is vulnerable to stress-related injury. Describing for the first time how trabecular density and microarchitecture differ between individuals with and without CPHP has the potential to fill an important evidence gap in our understanding of factors important to bone and entheseal health.

The aim of this study therefore, is to determine whether having CPHP is associated with calcaneal bone density and/or trabecular microarchitecture at the plantar calcaneus and at a mid-calcaneal reference site.

## Participants and methods

### Study design

Cross-sectional analysis of a case-control study using case status (presence of CPHP) as the exposure of interest.

### Setting & participants

Cases were recruited in southern Tasmania between November 2014 and May 2018 from general and specialist medical clinics, allied health practices, newspaper advertising, social media, sporting clubs and workplaces (hospitals and government departments). Sex- and age-matched control participants were recruited by random selection from the Tasmanian Electoral Roll (population 176,644, 2016) (one control:2 cases) from November 2016 to August 2018. This study was conducted in accordance with the principles of the Declaration of Helsinki and approved by the Tasmanian Health & Medical Human Research Ethics Committee (H0013616, 20 March, 2014). All participants provided written informed consent.

### Inclusion/Exclusion

Inclusion criteria for cases were: aged over 18 years, have CPHP defined as pain under the heel aggravated by ‘1^st^ step’ (pain under the heel on first step in the morning, or after a period of non-weightbearing rest) or pain aggravated by prolonged weightbearing, with symptoms present for at least 3 months. In the presence of bilateral heel pain, the most symptomatic heel was assessed. Control participants were matched for age (5-year brackets from 25–90 years) and sex and must never have had CPHP.

Both cases and controls were excluded if they had any contraindication to MRI, a history of previous foot fracture, ankle fracture (requiring cast or surgery) or orthopaedic foot surgery, current ankle pain, recent foot trauma or any other orthopaedic, congenital or painful lower limb condition that restricted mobility or activity in the preceding 3 months. Participants with peripheral vascular or central or peripheral neurological disease, including a current or recent history of lumbar radiculopathy, were also excluded. Cases who had a corticosteroid or any other injection, shockwave therapy or steroid iontophoresis within the previous 6 months were excluded.

### Sample size calculations

The case-control analysis is nested within a 12-month longitudinal study of cases. Sample size calculations for the longitudinal component of the study determined that 220 cases were required (assuming α = 0.05 (two-tailed) and 80% power for longitudinal hypotheses, with a loss to follow up of 10%). As previously published, we assumed controls would have values for key exposures seen in participants in community-based studies in our institution or from control participants in other CPHP studies [6]. Based on those values, and assuming power of 80%, and a two-tailed α of 0.05, we calculated that 100 controls were required to detect clinically important effect sizes that were comparable to or smaller than effect sizes reported in the CPHP, tendinopathy and osteoarthritis literature.

### Data collection

#### HR-pQCT scanning procedure

HR-pQCT scans were performed by the same experienced radiographer (KS) using a Scanco Xtreme CT I (Scanco Medical AG, Brüttisellen, Switzerland). Prior to scanning, daily calibration using manufacturer supplied reference phantoms was undertaken, according to Scanco protocol. The test foot of the participant was immobilised in the manufacturer provided carbon fibre lower leg cast for insertion into the scanner gantry. A custom-made Perspex plantar foot cradle was fixed to the plantar aspect of the inside of the cast to move the plantar surface of the calcaneus proximally into the scan zone. Standard Scanco Xtreme scan parameters were used; 60 kVp effective energy and 95 mA (collecting tube current) collecting 750 projections over a 180° rotation of the X-ray source, integration time of 100ms, FOV 126mm and image matrix 1536 x 1536, yielding an isotropic voxel of 82μm. Scans were assessed at the time for movement artefacts including horizontal streaking, loss of cortical contiguity or significant trabecular smearing [10], and were repeated if there was clear evidence of artefact.

#### Region of interest (ROI) selection and analysis

**Fig 1:**
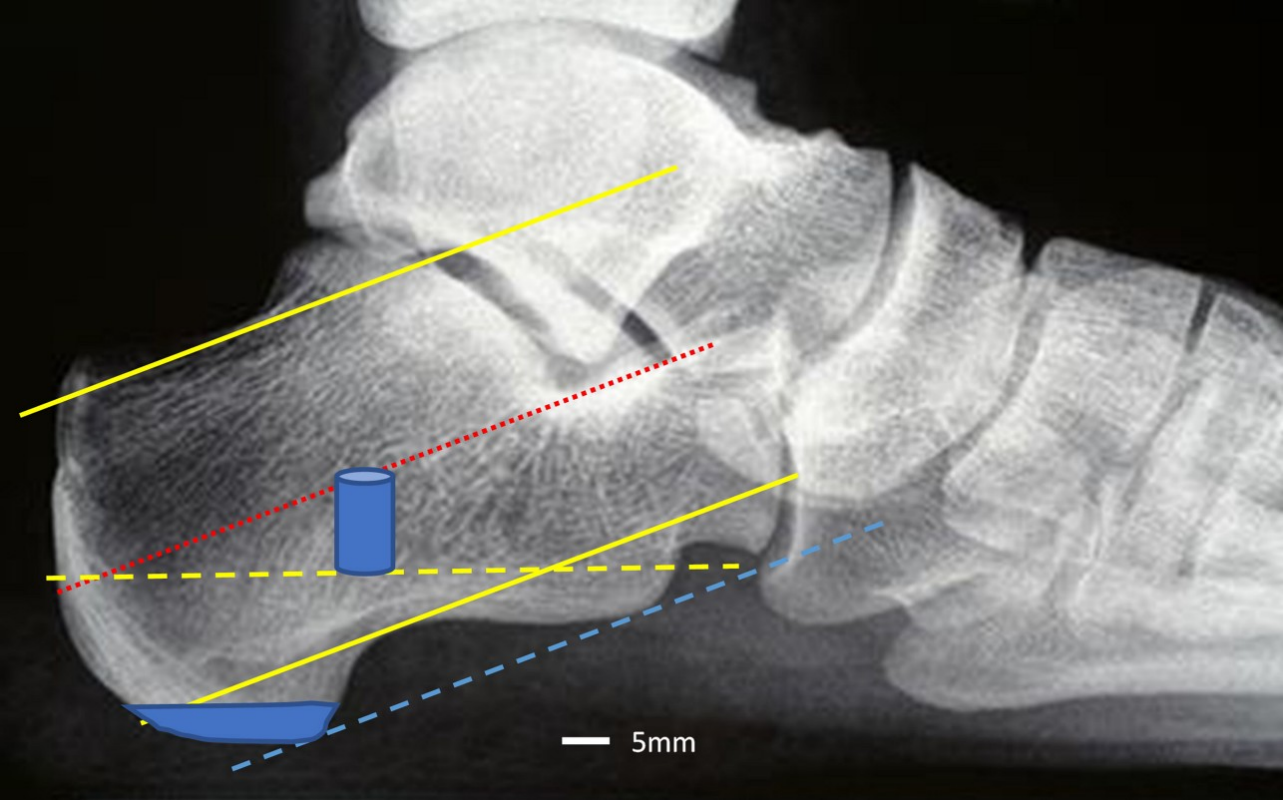
Location of mid-calcaneal and plantar ROI. Mid-calcaneal (cylinder) and plantar ROIs are shaded.

Plantar and mid-calcaneal regions were located on a scout image (**Fig 1**).

The plantar ROI captured the site of attachment of the plantar fascia on the weightbearing plantar tuberosity of the calcaneus. The plantar stack was initiated at the plantar most aspect of calcaneal bone. Two stacks were captured (220 slices) and the first 60 slices (~5mm) of the calcaneal tuberosity were contoured and analysed.

The mid-calcaneal ROI was positioned in a single mid-calcaneal stack half-way along the sagittal length of the calcaneus between its posterior and anterior-most margins (**Fig 1**). A plantar reference line, two parallel pitch lines hugging the superior and inferior waist of the calcaneus and a third parallel line bisecting these (representing the central pitch axis), were drawn. The posterior exit point of this last line located the inferior starting point of the mid-calcaneal stack. The mid-calcaneal ROI is cylindrical in shape measuring approximately 1cm^2^ by 1 stack high (110 slices/~9mm).

For each ROI, native Scanco analysis software (V6.5–3) was used to generate trabecular density (milligrams of hydroxyapatite/cubic centimetre (mg HA/cm^3^)), separation (mm), thickness (mm) and number (1/mm) measures, bone volume fraction (BV/TV %), and cortical density (mg HA/cm^3^), mean thickness (mm) and area (mm^2^) measures, as described previously [11–13]. Trabecular bone mineral density and trabecular number are calculated directly whereas trabecular thickness, separation and BV/TV are derived from these measurements. Bone volume fraction was derived from trabecular density assuming fully mineralised bone of 1200 mg HA/cm^3^.

Post scan processing was undertaken by a single blinded assessor for each region (plantar KS, mid-calcaneal AL) who placed and contoured all ROI’s. This process has been described previously [13,14] and includes semi-automatic edge contouring, visual checking for errors, and automatic bone compartment segmentation based on binarization with a fixed threshold.

Intra-study intra-class correlation co-efficients (ICCs) assessing the repeatability of BV/TV measures was excellent for both the plantar (ICC = 0.99 (3,1), n = 30) and mid-calcaneal ROIs (ICC = 0.90 (3,1), n = 30).

Scan quality was assessed by visual assessment of the plantar stack image and consideration of the presence of ring artefacts and signal to noise ratio (graininess). Scans were graded ordinally as (3) poorer quality (ring artefacts approaching but outside of ROI and/or higher noise), (2) fair (minor ring artefacts and/or moderate noise) and (1) good (no or minimal ring artefact, low noise). Repeatability for assessing scan quality was good (weighted kappa = 0.82, percent agreement 94%, n = 50).

Clinical measures were taken in a single session, in the same order, by the same experienced physiotherapist. Shoes, socks and lower leg clothing were removed. Measurement and reliability of clinical measures for height, weight, BMI, ankle plantarflexor strength, and physical activity by accelerometry have been summarised previously [6].

In brief, height was measured to the nearest 0.1cm using a stadiometer. Weight was measured to the nearest 0.1kg by a single set of calibrated scales (A&D Medical UC321-PL, Adelaide, South Australia), and body mass index calculated (weight(kg)/ht(m)^2^). Maximum isometric ankle plantarflexor strength was measured in sitting as the highest score from three attempts with the lower limb strapped by inelastic belt about the knee to a digital scale (Excell GW, Taiwan; sensitivity 0.05kg) (study ICC_3,1_ = 0.96, n = 18) [15]. Physical activity was measured by uniaxial accelerometer worn at the waist (Actigraph GT1M, Fort Walton Beach, Florida) monitored over 7 consecutive days [16]. Participant’s data were included if worn for a minimum of 5 days with at least 10 hours of monitored data. Wear-time was cross-checked with the use of a home diary. Non-wear time was defined by an interval of 55–60 consecutive minutes of zero activity intensity counts, with allowance for 1–2 min of counts between 0 and 100 [17]. We measured steps per day and mean counts per minute (CPM), classifying physical activity as minutes spent in moderate to vigorous (MVPA), light and sedentary activity. Separate thresholds were chosen for adults aged 18–64 years [18], and older adults aged 65–85 years [19,20], namely: MVPA (≥1,952 CPM/≥1065 CPM, respectively), light physical activity (100–1951 CPM/50-1064 CPM) and sedentary time (<100 CPM/<50 CPM). Data were downloaded using Actilife version 6 (Actigraph, Pensacola, FL).

Questionnaires recorded footwear choices, time spent in standing, age, sex, menopausal status, level of education, employment, smoking history and co-morbidities (diabetes or rheumatological disease). Section two of the Foot Health Status Questionnaire (FHSQ) was used to assess foot function (Cronbach’s alpha 0.86, ICC = 0.92) [21]. Quality of life was measured with the Australian Quality of Life scale, AQoL-6D [22] (Cronbach’s alpha 0.94, ICC = 0.85 to 0.88), (http://www.aqol.com.au/aqolquestionnaires/56.html).

### Statistical analysis

Characteristics of cases and controls were compared using descriptive statistics. Univariable and then multivariable linear regression models were used to estimate the association between CPHP status and QCT bone outcomes. We adjusted for age and sex, and then for other potential factors affecting bone including BMI, ankle plantarflexor strength and physical activity, based on biological plausibility. We assessed for effect modification by CPHP with these variables, and retained interaction terms where significant (p<0.05). Coefficients are reported as standardized betas with the independent variables centred and standardized, giving a change in outcome per SD variation in exposure.

Sensitivity analyses were conducted to assess the effect of i) scan quality (by omitting lower quality (grade 3) scans), and ii) removal of outliers on parameter estimates and statistical inference. Standard model fit checks included lowess and design variable plots, and assessment of best power fit by fractional polynomials. Assessment of residuals, link and multi-collinearity checks were also undertaken. All analyses were performed using Stata 16 (Stata Corp., College Station 16, TX, USA).

#### Patient and public involvement

No patients or the public were involved in the planning, design or the implementation of this study. Patients were not invited to contribute to interpretation of the results, nor the writing or editing of this document.

## Results

**Fig 2:**
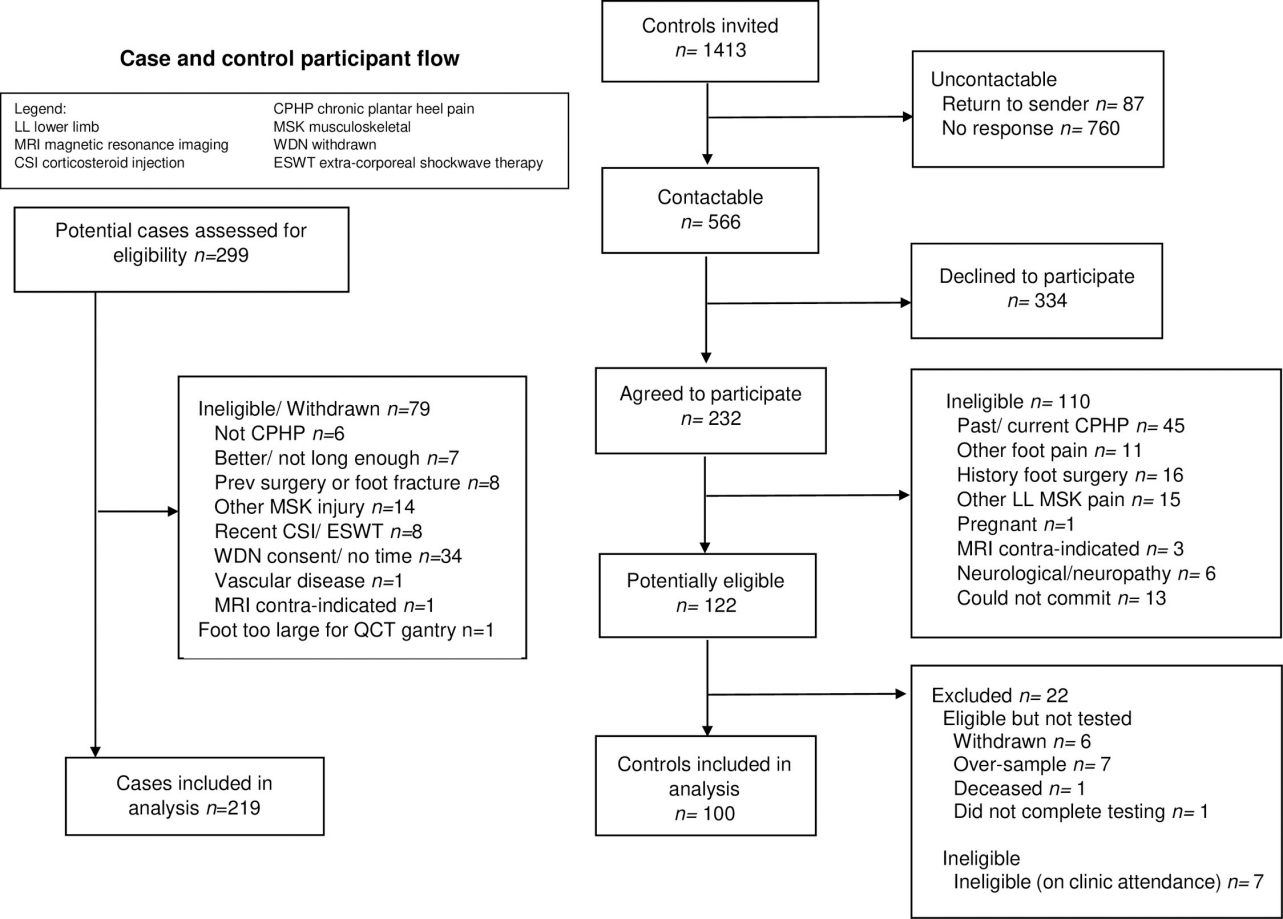
Case and control participant flow.

We tested 220 eligible cases from 299 potential cases screened, as previously described [6]. Of these, 219 who had HR-pQCT scans of their affected foot were included in this analysis. Of 566 contactable potential control participants, 232 agreed to participate. One-hundred and ten controls were excluded before consent, a further 22 withdrew or were excluded after consent but before testing was completed, leaving 100 control participants in the analysis (**Fig 2**).

Data availability for exposures was similar for cases and controls, ranging between 95–100%. In-session quality checking resulted in repeating scans for 12 participants (3 cases/9 controls, 4 men/8 women, 8 plantar stack rescans/4 mid-calcaneal). As previously reported cases and controls reported similar levels of comorbidities such as inflammatory disease, diabetes and high cholesterol, similar levels of self-reported menopause and smoking rates and comparable levels of physical activity [6] (**Table 1**). Cases had higher BMI and waist girth, and lower ankle plantarflexor strength, QOL and foot-related function than controls. Use of prescribed bone density medication was low for both cases (1.7%) and controls (3%).

**Table 1:** Characteristics of case and controls.

		Case (n = 219)*	Control (n = 100)*
Age (years)	Male	58.5 (12.1)	59.2 (12.5)
	Female	52.2 (11.5)	52.0 (11.6)
Female %(n)		59.6 (131)	60 (60)
Menopause^a^ %(n)		36.6 (48/131)	41.7 (25/60)
On HRT^a^ %(n)		6.9 (9/131)	8.3 (5/60)
Smoke- ever %(n)		33 (73)	28 (28)
Smoke- current %(n)		4.6 (10))	7 (7)
Diabetes %(n)		3.7 (8)	3 (3/99)
Inflammatory disease %(n)		8.2 (18)	6 (6)
High Cholesterol %(n)		25 (55)	30 (30)
Prescribed bone density medications %(n)		1.9 (4/214)	3 (3/99)
**Physical activity**			
Accelerometry (median, IQR)			
Average steps/day^b^		7918 (6148, 9903)	8373 (6080, 10205)
Moderate-to-vigorous (average min/day)^c^		38 (18, 61)	42 (20, 58)
Sedentary (average min/day)^d^		491 (437, 545)	502 (443, 561)
**Quality of life & function**			
AQOL-6D^e^		76.4 (10.8)	86.38 (6.9)
FHSQ function^f^		65.74 (27.8)	99.13 (3.3)
**Clinical measures**			
BMI (kg/m^2^)		29.14 (5.4)	27.63 (5.6)
Waist girth (cm)		97.43 (13.9)	90.82 (15.1)
Fat mass (%)		34.24 (8.9)	33.86 (8.2)
Foot posture index (FPI)		2.71 (3.9)	3.07 (3.7)
Ankle dorsiflexion ROM, knee flexed (deg)		43.26 (6.7)	44.15 (5.5)
Ankle plantarflexor strength (kg)		90.49 (23.8)	98.82 (26.4)
1^st^ MTP extension ROM (deg)		70.28 (15.1)	74.78 (14.4)

* values are Mean (SD), unless specified otherwise.

^a^ % of females only.

^b^case n = 207, control n = 96.

^c^case n = 211, control n = 96.

^d^case n = 211, control n = 96.

^e^Assessment of Quality of Life-6 Dimension scale, 0–100, higher is better.

^f^ Foot Health Status Questionnaire (weighted), 0–100, higher = better function.

MTP metatarsophalangeal, ROM Range of motion.

Bone density and microstructural parameters were similar in cases and controls at the mid-calcaneal site (**Table 2**). There were small differences at the plantar calcaneus with greater BV/TV and trabecular bone density, trabecular thickness and lower trabecular separation, in cases compared to controls.

**Table 2:** Bone indices.

**Mid calcaneal ROI**	Case (n = 219)		Control (n = 100)	
	mean	sd	mean	sd
Trabecular density (mg HA/cm^3^)	163	41	165	44
Bone volume fraction (BV/TV)	0.14	0.03	0.14	0.04
Trabecular number (/mm)	3.95	0.20	3.93	0.20
Trabecular separation (mm)	0.219	0.017	0.220	0.017
Trabecular thickness (mm)	0.034	0.008	0.035	0.009
**Plantar ROI**	Case (n = 219)		Control (n = 100)	
	mean	sd	mean	sd
Trabecular density (mg HA/cm^3^)	**356**	**37**	**345**	**47**
Bone volume fraction (BV/TV)	**0.30**	**0.03**	**0.29**	**0.04**
Trabecular number (/mm)	3.95	0.15	3.92	0.16
Trabecular separation (mm)	**0.178**	**0.012**	**0.182**	**0.015**
Trabecular thickness (mm)	**0.075**	**0.008**	**0.073**	**0.009**
Cortical density (mg HA/cm^3^)	641	69	647	79
Mean cortical thickness (mm)	0.846	0.41	0.897	0.55
Mean cortical area (mm^2^)	57.9	29.5	57.6	36.1

Bold denotes p for case-control comparison <0.05, t-test for continuous data.

ROI region of interest, sd standard deviation.

Univariable associations of CPHP and other factors with bone outcomes are given in Table 3, with those associations having P<0.05 indicated in bold. Having CPHP was associated with higher trabecular density, BV/TV and trabecular thickness and lower trabecular separation in the plantar ROI but was not associated with any mid-calcaneal bone outcome (**Table 3, all p<0.05, ranging from 0.012 to 0.041)**. At both ROIs, BMI and ankle plantarflexor strength were positively associated with trabecular bone density, BV/TV and thickness, negatively associated with trabecular separation, and positively associated with trabecular number except for ankle plantarflexor strength at the mid-calcaneal ROI. Female sex was negatively associated with all bone outcomes at both ROIs except for a positive association with trabecular separation, and no association with trabecular thickness. Age was negatively associated with trabecular density, BV/TV and thickness outcomes in the mid-calcaneum. In the plantar region age was not associated with trabecular density, but positively associated with trabecular number and negatively associated with trabecular thickness.

**Table 3:** Univariable results for mid-calcaneal and plantar ROI, standardized co-efficients(se)^a^^,^^b^.

Mid-calcaneal ROI	Trabecular density (mg HA/cm^3^)	BV/TV (%)	Trabecular thickness (mm)	Trabecular number (/mm)	Trabecular separation (mm)	
Case status^c^	-0.9 (2.4)	-0.001 (0.002)	-0.000 (0.001)	0.011 (0.011)	-0.000 (0.001)	
BMI	**11.6** (2.3)	**0.010** (0.002)	**0.002** (0.001)	0.021 (0.011)	**-0.004** (0.001)	
Age	**-10.3** (2.3)	**-0.009** (0.002)	**-0.002** (0.001)	0.008 (0.011)	0.002 (0.001)	
Female sex^d^	**-5.2** (2.3)	**-0.004** (0.002)	0.000 (0.001)	**-0.128** (0.009)	**0.008** (0.001)	
Ankle PF strength	**8.7** (2.3)	**0.007** (0.002)	**0.001** (0.001)	**0.088** (0.099)	**-0.007** (0.051)	
MVPA^e^	1.3 (2.4)	0.001 (0.002)	0.000 (0.001)	**0.035** (0.011)	**-0.002** (0.001)	
Plantar ROI	Trabecular density	BV/TV	Trabecular thickness	Trabecular number	Trabecular separation	Cortical density (mg HA/cm^3^)
Case status^c^	**5.4** (2.3)	**0.005** (0.002)	**0.001** (0.001)	0.013 (0.009)	**-0.002** (0.001)	-2.7 (4.1)
BMI	**11.7** (2.1)	**0.010** (0.002)	**0.002** (0.001)	**0.046** (0.008)	**-0.005** (0.001)	1.1 (4.1)
Age	-3.4 (2.3)	-0.003 (0.002)	**-0.001** (0.001)	**0.025** (0.009)	-0.000 (0.001)	**-19.1** (3.9)
Female sex^d^	**-7.7** (2.2)	**-0.006** (0.002)	0.000 (0.001)	**-0.093** (0.007)	**0.006** (0.001)	**-9.8** (4.0)
Ankle PF strength	**10.3** (2.2)	**0.009** (0.002)	**0.001** (0.001)	**0.059** (0.008)	**-0.005** (0.001)	**12.4** (4.0)
MVPA^e^	-1.1 (2.4)	-0.001 (0.002)	-0.001 (0.001)	**0.023** (0.009)	-0.001 (0.001)	2.0 (4.2)

ROI region of interest, BV/TV bone volume/total volume, BMI body mass index, PF plantarflexor.

^a^Univariable linear regression model, standardized X co-efficient/unstandardized Y (standard error). All n = 319 unless stated.

^b^Bold denotes statistically significant with p<0.05.

^c^case = 1, control = 0.

^d^female = 1, male = 0.

^e^ MVPA, moderate to vigorous physical activity, average minutes per day, by accelerometry, n = 306.

**Fig 3:**
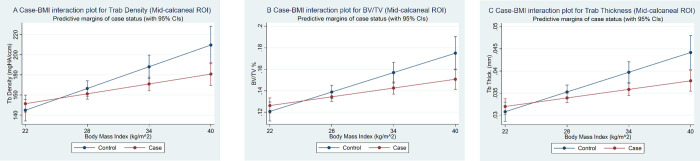
(A) Case-BMI interaction plots for mid-calcaneal ROI trabecular density, (B) BV/TV and (C) trabecular thickness. Bone outcomes plotted from the 10th to 90th percentile of BMI.

**Fig 4:**
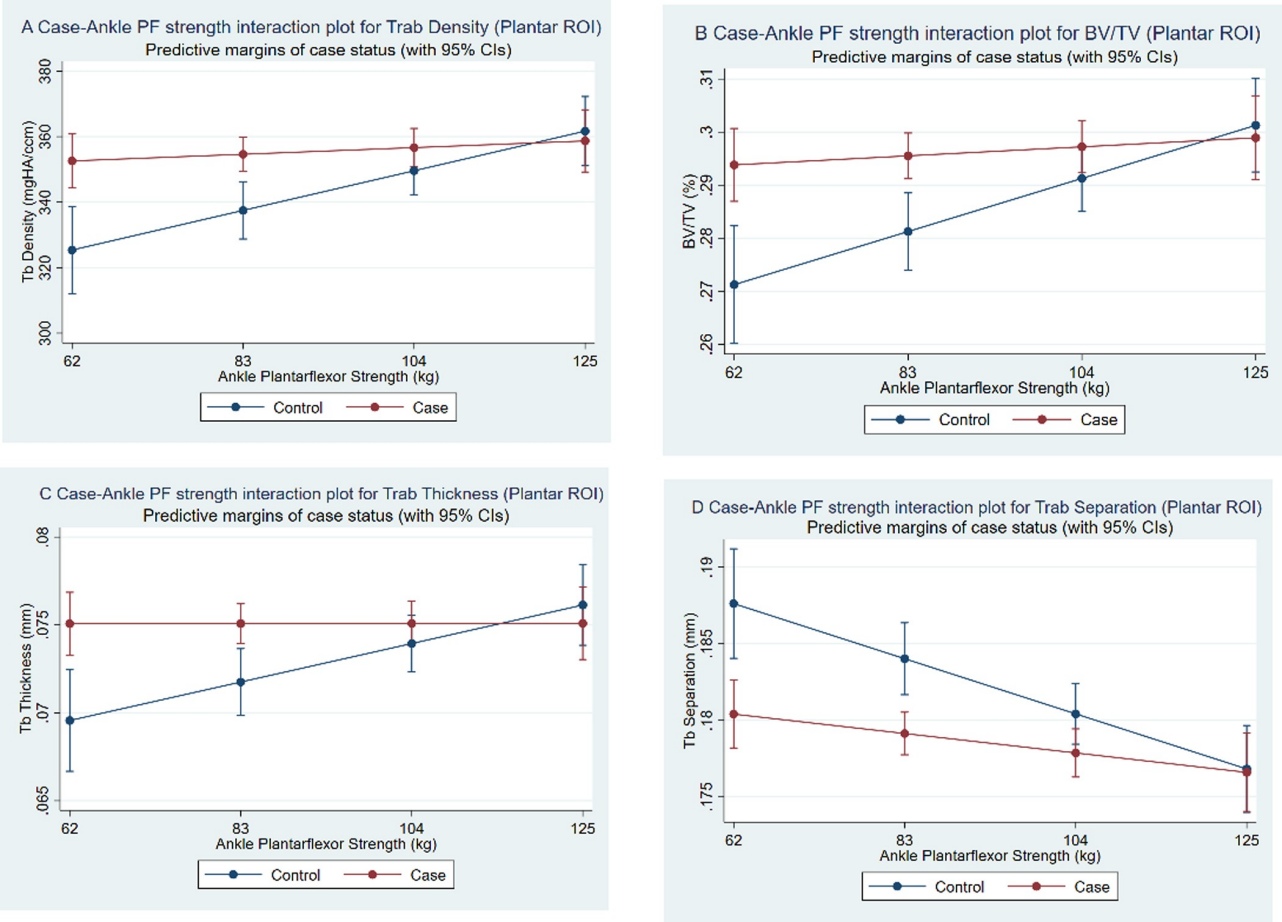
(A) Case-ankle plantarflexor strength interaction plot for plantar ROI trabecular density, (B) BV/TV, (C) thickness and (D) separation. Bone outcomes plotted from the 10th to 90th percentile of ankle plantarflexor strength.

Associations of CPHP status with bone outcomes were largely unchanged in an adjusted main effects model (**S1 Table**) but there were significant interaction terms in the final multivariable model, with those associations having p<0.05 highlighted in bold (**Table 4, all interaction terms p<0.05, ranging from 0.006 to 0.019**). The positive association of BMI with mid-calcaneal trabecular density, BV/TV and thickness was less in cases compared to controls (**Fig 3A–3C**). In the plantar ROI, the positive association of ankle plantarflexor strength with plantar trabecular density, BV/TV and thickness (**Fig 4A–4C**), and the negative association with trabecular separation **(Fig 4D**), was less in cases compared to controls. Differences between cases and controls in these plantar bone indices are greater at lower levels of ankle plantarflexor strength. The associations of factors which did not show effect modification by CPHP status were similar to those in univariable models, except that ankle plantarflexor strength in the mid-calcaneal ROI and age in the plantar ROI had lower coefficients for most bone outcomes in the multivariable model.

**Table 4:** Multivariable results for mid-calcaneal and plantar ROI, standardized co-efficients(se)^a^^,^^b^.

Mid-calcaneal ROI	Trabecular density^d^ (mg HA/cm^3^)	BV/TV (%)^d^	Trabecular thickness (mm)^d^	Trabecular number (/mm)^d^	Trabecular separation (mm)^e^	
Case status^c^	**-6.6** (4.8)	**-0.006** (0.004)	**-0.002** (0.001)	0.029 (0.018)	-0.001 (0.002)	
BMI	**19.9** (3.8)	**0.017** (0.003)	**0.004** (0.001)	**0.021** (0.008)	**-0.005** (0.001)	
BMI*Case interaction	**-10.8** (4.6)	**-0.009** (0.004)	**-0.002** (0.001)	-	-	
APFS	-0.05 (2.7)	0.000 (0.002)	-0.000 (0.001)	**0.036** (0.010)	**-0.003** (0.001)	
Age	**-13.1** (2.4)	**-0.011 (0.002)**	**-0.003 (0.000)**	-0.015 (0.009)	**0.004** (0.001)	
Female sex	**-9.0** (2.6)	**-0.008 (0.002)**	-0.002 (0.001)	**-0.118** (0.010)	**0.008** (0.001)	
MVPA	-	-	-	-	**-0.002** (0.001)	
Plantar ROI	Trabecular density (mg HA/cm^3^)^f^	BV/TV (%)^f^	Trabecular thickness (mm)^f^	Trabecular number (/mm)^g^	Trabecular separation (mm)^f^	Cortical density (mg HA/cm^3^)^f^
Case status^c^	**12.3** (4.6)	**0.010** (0.004)	**0.002** (0.001)	0.027 (0.014)	**-0.004** (0.001)	-7.3 (8.6)
APFS	**14.4** (3.8)	**0.012** (0.003)	**0.003** (0.001)	**0.026** (0.008)	**-0.004** (0.001)	-0.9 (4.8)
APFS*Case interaction	**-11.9** (4.4)	**-0.010** (0.004)	**-0.003** (0.001)	-	**0.003** (0.001)	-
BMI	**10.4** (2.1)	**0.009** (0.002)	**0.001** (0.001)	**0.047** (0.007)	**-0.004** (0.001)	2.8 (3.9)
Age	-3.7 (2.4)	-0.003 (0.002)	-0.001 (0.001)	0.007 (0.008)	0.001 (0.001)	**-23.8** (4.4)
Female sex	**-6.4** (2.5)	**-0.005** (0.002)	0.000 (0.001)	**-0.084** (0.008)	**0.005** (0.001)	**-16.6** (4.7)
MVPA	-	-	-	0.009 (0.007)	-	-

ROI region of interest, BV/TV bone volume/total volume, BMI body mass index, APFS ankle plantarflexor strength, MVPA moderate to vigorous physical activity.

^a^Multivariable linear regression model, standardized X co-efficients, unstandardized Y (standard error).

^b^Bold denotes statistically significant with p<0.05.

^c^case = 1, control = 0.

^d^Adjusted for age, sex & ankle plantarflexor strength, n = 319.

^e^Adjusted for age, sex, ankle plantarflexor strength and physical activity (MVPA), n = 306.

^f^Adjusted for age, sex, & BMI, n = 319.

^g^Adjusted for age, sex, BMI & physical activity (MVPA), n = 306.

### Sensitivity analyses

Scan quality was lower in 83 participants (16 controls and 67 cases) who were mostly men (76/83). A longer foot was associated with scan quality classification (foot length: good (1) 11.2 (0.7) cm, fair (2) 11.7 (0.8) cm, poor (3)12.3 (0.7) cm). Sensitivity analysis omitting lower quality (grade 3) resulted in only small changes (~6%) in mid-calcaneal BV/TV and trabecular density interaction term co-efficients (**S2 Table**) (p-values = 0.050 and 0.051 respectively). Other interactions were similar. The only substantial changes in coefficients for other factors were a decrease in effect size for sex for trabecular density and BV/TV and an increase for MVPA with plantar trabecular number. Sensitivity analyses omitting influential observations did not otherwise result in significant changes in parameter estimates for bone outcomes in either ROI (S3 Table).

## Discussion

Having CPHP modified the effect of BMI and ankle plantarflexor strength on trabecular bone outcomes in the mid- and plantar calcaneus, respectively. Higher BMI had beneficial associations with bone outcomes (higher trabecular density, thickness and BV/TV) in the mid-calcaneal region in controls but not in cases. Higher ankle plantarflexor strength had beneficial associations with bone outcomes (higher trabecular density, thickness and BV/TV and lower trabecular separation) in the plantar region in controls but not in cases. The reasons for differences in associations between cases and controls are not clear, but could include being due to a stress-related entheseal reaction in cases, altered physical loading strategies due to pain, or other mechanisms associated with pain, systemic inflammation or neurogenic factors. Confirmation of effects of CPHP on bone health in longitudinal studies is needed.

The finding that having CPHP modifies the association between ankle plantarflexor strength and plantar trabecular bone is new. The ankle plantarflexors transmit large forces through the posterior and plantar calcaneus to the plantar fascia [5], creating a plausible mechanism for local bone stimulation as seen in controls. As seen in **Fig 4A–4D**, trabecular density, BV/TV and thickness are higher and trabecular separation is lower in cases than controls at low levels of ankle plantar flexor strength but similar at higher levels. Cases appear to respond differently to locally applied active loads. Cases may have a stimulatory bone response to entheseal stress that over-rides or precedes the normal stimulatory effect of locally applied forces by the Achilles/plantar fascia complex seen in controls. Increased bone turnover is a common feature of bone under stress, and parallels can be drawn to similar reactions observed in subchondral bone in OA [23]. The higher prevalence of plantar enthesophytes in CPHP [4] supports this. These are thought to develop in response to entheseal degenerative change [3] and hypothesized to serve a load sharing, adaptive function in response to such stress [3,24]. Another feature of this degenerative process which might add bone mineral to the plantar heel is calcification of the large fibrocartilaginous enthesis attachment of the plantar fascia [3]. The interaction plots (**Fig 4A–4D**) suggest that there could be a ceiling beyond which the capacity for further bone adaptation, despite increased local loads, is exhausted. Overall, these findings may indicate disturbed coupling of the effects of actively applied local loads to the plantar calcaneus. Conversely, ankle plantarflexor strength was not consistently associated with mid-calcaneal bone outcomes, which is perhaps expected as this ROI is situated well away from the trabecular pathways associated with Achilles-plantar fascia force transmission.

The finding that CPHP modifies the effect of BMI on mid-calcaneal bone outcomes is also novel. This may be explained by differences in calcaneal loading. BMI has a stimulatory effect on bone likely via mechanical loading pathways [25]. Gait studies indicate that cases load the heel differently [26], which may reduce the weightbearing stimulus in the mid-calcaneum. A second possibility for the differences in bone outcomes could include metabolic and/or meta-inflammatory mechanisms. BMI is correlated with waist girth, which is itself strongly associated with CPHP [6]. Waist girth is an important proxy for cardiometabolically active central adiposity, which is known for its cytokine-producing systemic meta-inflammatory effects [27]. Osteoclast function in trabecular bone is sensitive to cytokine activity [28] associated with systemic inflammation. The positive effect of body mass on bone may be countered in cases by the negative effect of central adiposity, especially in older people [29]. A final consideration is the action of local peripheral nerve tissue which is important in bone homeostasis [30]. As a pain condition, sensory afferents associated with nociception release osteotrophic neurotransmitters such as CGRP, substance p and glutamate [31], which may have a direct effect on bone metabolism. Regardless, these findings underscore that there are consequences for bone distant to the enthesis in CPHP.

The effects of age and sex were generally consistent with their known effect on trabecular bone with both female gender and increasing age associated with a decrease in bone structure. An exception to this was that greater age was consistently not associated with bone outcomes in the plantar ROI. Ageing is associated with increased mineralization of the fibrocartilage transitional zone and subchondral plate thickening at the enthesis (in the presence of cortical thinning) potentially countering age-related trabecular bone loss in this region [32]. It may also be that at this site, the usual negative influence of age may be countered by the stimulatory effect of locally applied plantar fascia-ankle plantarflexor forces.

Previous HR-pQCT data for the calcaneus are very sparse, with two studies of 18 and 5 people respectively [7,8]. Comparisons with our study is difficult as the study samples are different, being young athletic males [7] and aged cadaveric/ex vivo women [8]. They analysed different regions within the calcaneus [7] or summed wider regions of interest [8]. These differences make it difficult to generalize results and technical parameters across studies and indicate that more in vivo work across a greater range of age, sex and calcaneal sites is required. We are re-assured however by the consistency of our results across bone outcomes and between ROIs. Furthermore, in unadjusted analyses age, sex and BMI follow known biologic patterns for their effect on bone and provides further evidence of the validity of our measures.

### Limitations

This is the first study to compare bone microstructure and density outcomes in participants with CPHP. Its strengths include its large sample size, extensive covariate set, and the comparison with population-derived controls, however there are limitations. Our results describe cross-sectional differences in bone outcomes between cases and controls, and cannot assign causation. Longitudinal studies of these bone outcomes would provide further insight on the relationship between these findings and CPHP. Selection bias is possible. This was minimized in controls by recruiting randomly from the electoral role and we maximised the representativeness of cases by recruiting from a range of sources; print and social media, community clubs and organisations, healthcare practices and from word of mouth. Cases and controls were recruited from the same local catchment area, and were matched for the key demographics of age and sex. Scan quality may impact findings. However, a sensitivity analysis removing poorer quality scans had little effect on the magnitude of associations at both ROIs for our primary exposure of interest (case status) and its interaction effects, and our overall conclusions remain unchanged. The analysis did alter the coefficient for sex in both ROIs, probably because poorer quality scans were mostly in males.

## Conclusion

Having chronic plantar heel pain may have consequences for calcaneal bone structure by modifying associations of BMI and ankle plantarflexor strength with bone outcomes at the mid- and plantar calcaneus respectively. The beneficial associations of BMI with bone outcomes seen in controls in the mid-calcaneal region and with greater ankle plantarflexor strength in the plantar calcaneal region is either reduced or absent in cases. The reasons for differences in associations between cases and controls are not clear, but could include a bone response to entheseal stress in cases, altered physical loading strategies in response to pain or other mechanisms associated with pain, systemic inflammation and neurogenic factors. These findings require further validation and ideally confirmation with longitudinal data.

## Supporting information

S1 Table Main effects model, standardized co-efficients (se)^a^. ROI region of interest, BMI body mass index, PF plantarflexor, BV/TV bone volume fraction, MVPA moderate to vigorous physical activity. ^a^Multivariable linear regression model, standardized X co-efficients/unstandardized Y (standard error). Bold denotes statistically significant with p<0.05. ^b^case = 1, control = 0. ^c^Adjusted for age & sex, n = 319. ^d^Adjusted for age, sex and physical activity (MVPA), n = 306.
(DOCX)

S2 Table Sensitivity analysis for scan quality; omitting poorer quality (grade 3) scans, standardised co-efficients (se)^a^. ROI region of interest, BV/TV bone volume/total volume, BMI body mass index, APFS ankle plantarflexor strength, MVPA moderate to vigorous physical activity. ^a^Multivariable linear regression, standardized X/unstandardized Y co-efficients (standard error). Bold denotes statistically significant with p<0.05, Underline = changed significance, ^b^case = 1, control = 0, ^c^Moderate to vigorous physical activity, average minutes per day, by accelerometry. ^d^Adjusted for age, sex & ankle plantarflexor strength, n = 236 ^e^Adjusted for age, sex, ankle plantarflexor strength and physical activity (MVPA), n = 227. ^f^Adjusted for age, sex, & BMI, n = 236. ^g^Adjusted for age, sex, BMI & physical activity (MVPA), n = 227.
(DOCX)

S3 Table Sensitivity analysis; omitting influential observations, standardised co-efficients (se)^a^. ROI region of interest, BV/TV bone volume/total volume, BMI body mass index, APFS ankle plantarflexor strength, MVPA moderate to vigorous physical activity. ^a^Multivariable linear regression, standardized X/unstandardized Y co-efficients (standard error). Bold denotes statistically significant with p<0.05. ^b^case = 1, control = 0. ^c^Moderate to vigorous physical activity, average minutes per day, by accelerometry. ^d^Adjusted for age, sex & ankle plantarflexor strength. ^e^Adjusted for age, sex, ankle plantarflexor strength and physical activity (MVPA). ^f^Adjusted for age, sex, & BMI. ^g^Adjusted for age, sex, BMI & physical activity (MVPA).
(DOCX)
