# Supplementary material for: Chronic plantar heel pain modifies associations of ankle plantarflexor strength and body mass index with calcaneal bone density and microarchitecture
Source: PLoS One. 2021 Dec 9;16(12):e0260925. doi: 10.1371/journal.pone.0260925 (PMC8659683; doi:10.1371/journal.pone.0260925)
Supplement: S1 Table — ROI region of interest, BMI body mass index, PF plantarflexor, BV/TV bone volume fraction, MVPA moderate to vigorous physical activity. aMultivariable linear regression model, standardized X co-efficients/unstandardized Y (standard error). Bold denotes statistically significant with p<0.05. bcase = 1, control = 0. cAdjusted for age & sex, n = 319. dAdjusted for age, sex and physical activity (MVPA), n = 306. (DOCX) [file pone.0260925.s001.docx]

**Supplementary Table 1** Main effects model, standardized co-efficients (se)^a, b^

| Mid-calcaneal ROI | Trabecular density (mg HA/cm^3^)^d^ | BV/TV (%)^d^ | Trabecular thickness (mm)^d^ | Trabecular number (/mm)^d^ | Trabecular separation (mm)^e^ |  |
| --- | --- | --- | --- | --- | --- | --- |
| Case status^c^ | -2.5 (2.2) | -0.002 (0.002) | -0.001 (0.001) | 0.014 (0.008) | -0.000 (0.001) |  |
| BMI | **12.5** (2.2) | **0.010** (0.002) | **0.003** (0.000) | **0.021** (0.008) | **-0.005** (0.001) |  |
| Ankle PF strength | 0.5 (2.6) | 0.000 (0.002) | -0.000 (0.001) | **0.036** (0.010) | **-0.003** (0.001) |  |
| Age | **-12.9** (2.4) | **-0.011** (0.001) | **-0.003** (0.001) | -0.015 (0.009) | **0.004** (0.001) |  |
| Female sex | **-8.9** (2.6) | **-0.007** (0.002) | -0.001 (0.001) | **-0.118** (0.010) | **0.008** (0.001) |  |
| MVPA | - | - | - | - | **-0.002** (0.001) |  |
| Plantar ROI | Trabecular density (mg HA/cm^3^)^d^ | BV/TV (%)^d^ | Trabecular thickness (mm)^d^ | Trabecular number (/mm)^e^ | Trabecular separation (mm)^d^ | Cortical density (mg HA/cm^3^)^d^ |
| Case status^c^ | **5.0** (2.1) | **0.004** (0.002) | 0.001 (0.001) | 0.013 (0.007) | **-0.002** (0.001) | -3.4 (4.0) |
| BMI | **10.8** (2.1) | **0.009** (0.002) | **0.001** (0.001) | **0.047** (0.007) | **-0.004** (0.001) | 2.8 (3.9) |
| Ankle PF strength | **6.7** (2.6) | **0.006** (0.002) | 0.001 (0.001) | **0.026** (0.008) | **-0.003** (0.001) | -0.9 (4.8) |
| Age | -3.7 (2.4) | -0.003 (0.002) | -0.001 (0.001) | 0.007 (0.008) | 0.001 (0.001) | **-23.8** (4.4) |
| Female sex | **-6.3** (2.5) | **-0.005** (0.002) | 0.000 (0.001) | **-0.084** (0.008) | **0.005** (0.001) | **-16.6** (4.7) |
| MVPA | - | - | - | 0.009 (0.007) | - | - |

ROI region of interest, BMI body mass index, PF plantarflexor, BV/TV bone volume fraction, MVPA moderate to vigorous physical activity

^a^Multivariable linear regression model, standardized X co-efficients/ unstandardized Y (standard error)

^b^Bold denotes statistically significant with p<0.05

^c^case =1, control=0

^d^Adjusted for age & sex, n=319

^e^Adjusted for age, sex and physical activity (MVPA), n=306
