# Supplementary material for: Chronic plantar heel pain modifies associations of ankle plantarflexor strength and body mass index with calcaneal bone density and microarchitecture
Source: PLoS One. 2021 Dec 9;16(12):e0260925. doi: 10.1371/journal.pone.0260925 (PMC8659683; doi:10.1371/journal.pone.0260925)
Supplement: S2 Table — ROI region of interest, BV/TV bone volume/total volume, BMI body mass index, APFS ankle plantarflexor strength, MVPA moderate to vigorous physical activity. aMultivariable linear regression, standardized X/unstandardized Y co-efficients (standard error). Bold denotes statistically significant with p<0.05, Underline = changed significance, bcase = 1, control = 0, cModerate to vigorous physical activity, average minutes per day, by accelerometry. dAdjusted for age, sex & ankle plantarflexor strength, n = 236 eAdjusted for age, sex, ankle plantarflexor strength and physical activity (MVPA), n = 227. fAdjusted for age, sex, & BMI, n = 236. gAdjusted for age, sex, BMI & physical activity (MVPA), n = 227. (DOCX) [file pone.0260925.s002.docx]

**Supplementary Table 2**

Sensitivity analysis for scan quality; omitting poorer quality (grade 3) scans, standardised co-efficients (se)^a, b^

| Mid-calcaneal ROI | Trabecular density (mg HA/cm^3^)^e^ | BV/TV (%)^e^ | Trabecular thickness (mm)^e^ | Trabecular number (/mm)^e^ | Trabecular separation (mm)^f^ |  |
| --- | --- | --- | --- | --- | --- | --- |
| Case status^c^ | -7.1 (5.5) | -0.006 (0.005) | -0.002 (0.001) | 0.016 (0.022) | 0.001 (0.002) |  |
| BMI | **21.8** (4.4) | **0.018** (0.004) | **0.005** (0.001) | 0.020 (0.010) | **-0.005** (0.001) |  |
| BMI*Case interaction | -10.5 (5.3) | -0.009 (0.004) | **-0.002** (0.001) | - | - |  |
| APFS | 1.0 (3.0) | 0.001 (0.003) | -0.000 (0.001) | **0.042** (0.012) | **-0.003** (0.001) |  |
| Age | **-12.3** (2.9) | **-0.010** (0.002) | **-0.003** (0.001) | -0.019 (0.012) | **0.004** (0.001) |  |
| Female sex | -5.7 (3.0) | -0.005 (0.003) | -0.002 (0.002) | **-0.078** (0.012) | **0.005** (0.001) |  |
| MVPA^d^ | - | - | - | - | **-0.003** (0.001) |  |
| Plantar ROI | Trabecular density (mg HA/cm^3^)^g^ | BV/TV (%)^g^ | Trabecular thickness (mm)^g^ | Trabecular number (/mm)^h^ | Trabecular separation (mm)^g^ | Cortical density (mg HA/cm^3^)^g^ |
| Case status^c^ | **9.8** (5.6) | **0.008** (0.005) | **0.002** (0.001) | 0.020 (0.016) | **-0.003** (0.002) | -9.7 (10.4) |
| APFS | **13.2** (4.4) | **0.011** (0.004) | **0.002** (0.001) | **0.032** (0.009) | **-0.004** (0.001) | -2.1 (5.7) |
| APFS*Case interaction | **-13.4** (5.3) | **-0.011** (0.004) | **-0.003** (0.001) | - | **0.003** (0.001) | - |
| BMI | **10.8** (2.6) | **0.009** (0.002) | **0.001** (0.001) | **0.052** (0.008) | **-0.005** (0.001) | 1.9 (4.9) |
| Age | -3.9 (2.9) | -0.003 (0.002) | -0.001 (0.001) | 0.003 (0.009) | 0.001 (0.001) | **-27.3** (5.5) |
| Female sex | -2.7 (3.0) | -0.002 (0.003) | 0.001 (0.001) | **-0.054** (0.009) | **0.003** (0.001) | **-14.0** (5.7) |
| MVPA^d^ | - | - | - | **0.021** (0.008) | - | - |

ROI region of interest, BV/TV bone volume / total volume, BMI body mass index, APFS ankle plantarflexor strength, MVPA moderate to vigorous physical activity

^a^Multivariable linear regression, standardized X/ unstandardized Y co-efficients (standard error).

^b^Bold denotes statistically significant with p<0.05, Underline = changed significance

^c^case =1, control=0,

^d^Moderate to vigorous physical activity, average minutes per day, by accelerometry

^e^Adjusted for age, sex & ankle plantarflexor strength, n=236

^f^Adjusted for age, sex, ankle plantarflexor strength and physical activity (MVPA), n=227

^g^Adjusted for age, sex, & BMI, n=236

^h^Adjusted for age, sex, BMI & physical activity (MVPA), n=227
