# Supplementary material for: Chronic plantar heel pain modifies associations of ankle plantarflexor strength and body mass index with calcaneal bone density and microarchitecture
Source: PLoS One. 2021 Dec 9;16(12):e0260925. doi: 10.1371/journal.pone.0260925 (PMC8659683; doi:10.1371/journal.pone.0260925)
Supplement: S3 Table — ROI region of interest, BV/TV bone volume/total volume, BMI body mass index, APFS ankle plantarflexor strength, MVPA moderate to vigorous physical activity. aMultivariable linear regression, standardized X/unstandardized Y co-efficients (standard error). Bold denotes statistically significant with p<0.05. bcase = 1, control = 0. cModerate to vigorous physical activity, average minutes per day, by accelerometry. dAdjusted for age, sex & ankle plantarflexor strength. eAdjusted for age, sex, ankle plantarflexor strength and physical activity (MVPA). fAdjusted for age, sex, & BMI. gAdjusted for age, sex, BMI & physical activity (MVPA). (DOCX) [file pone.0260925.s003.docx]

**Supplementary Table 3**

Sensitivity analysis; omitting influential observations, standardised co-efficients (se)^a, b^

| Mid-calcaneal ROI | Trabecular density (mg HA/cm^3^)^e^ | BV/TV (%)^e^ | Trabecular thickness (mm)^e^ | Trabecular number (/mm)^e^ | Trabecular separation (mm)^f^ |  |
| --- | --- | --- | --- | --- | --- | --- |
| Case status^c^ | **-6.6 (**4.8**)** | **-0.007** (0.004) | **-0.002** (0.001) | 0.030 (0.018) | -0.001 (0.002) |  |
| BMI | **19.8** (3.9) | **0.018** (0.003) | **0.005** (0.001) | **0.022** (0.008) | **-0.005** (0.001) |  |
| BMI*Case interaction | **-10.7 (**4.7**)** | **-0.011 (**0.004**)** | **-0.003** (0.001) | - | - |  |
| APFS | -0.1 (2.7) | 0.001 (0.002) | -0.000 (0.001) | **0.036** (0.010) | **-0.003** (0.001) |  |
| Age | **-13.0** **(**2.5**)** | **-0.011 (0.002)** | **-0.003** (0.001) | -0.016 (0.009) | **0.003** (0.001) |  |
| Female sex | **-9.0 (**2.6**)** | **-0.007 (0.002)** | -0.001 (0.001) | **-0.240 (**0.020**)** | **0.008** (0.001) |  |
| MVPA^d^ | - | - | - | - | **-0.002** (0.001) |  |
| n | 313 | 316 | 315 | 313 | 295 |  |
| Plantar ROI | Trabecular density (mg HA/cm^3^)^g^ | BV/TV (%)^g^ | Trabecular thickness (mm)^g^ | Trabecular number (/mm)^h^ | Trabecular separation (mm)^g^ | Cortical density (mg HA/cm^3^)^g^ |
| Case status^c^ | **12.6** (4.7) | **0.009** (0.004) | **0.002** (0.001) | 0.026 (0.014) | **-0.004** (0.001) | -8.0 (8.6) |
| APFS | **13.7** (3.9) | **0.012** (0.003) | **0.003** (0.001) | **0.030** (0.008) | **-0.004 (**0.001**)** | -1.1 (4.8) |
| APFS*Case interaction | **-11.5 (**4.5**)** | **-0.010 (**0.004**)** | **-0.002 (**0.001**)** | - | **0.003** (0.001) | - |
| BMI | **10.3** (2.1) | **0.009** (0.002) | **0.001** (0.001) | **0.048** (0.007) | **-0.004** **(**0.001**)** | 2.8 (4.0) |
| Age | -3.4 (2.4) | -0.002 (0.002) | -0.001 (0.001) | 0.006 (0.007) | 0.001 (0.001) | **-24.1 (4.4)** |
| Female sex | **-6.5** (2.6) | **-0.005** (0.002) | 0.000 (0.001) | **-0.082** (0.008) | **0.010** (0.001) | **-16.2** **(4.7)** |
| MVPA^d^ | - | - | - | 0.009 (0.007) | - | - |
| n | 305 | 308 | 314 | 302 | 314 | 315 |

ROI region of interest, BV/TV bone volume / total volume, BMI body mass index, APFS ankle plantarflexor strength, MVPA moderate to vigorous physical activity

^a^Multivariable linear regression, standardized X/ unstandardized Y co-efficients (standard error)

^b^Bold denotes statistically significant with p<0.05

^c^case =1, control=0

^d^Moderate to vigorous physical activity, average minutes per day, by accelerometry

^e^Adjusted for age, sex & ankle plantarflexor strength

^f^Adjusted for age, sex, ankle plantarflexor strength and physical activity (MVPA)

^g^Adjusted for age, sex, & BMI

^h^Adjusted for age, sex, BMI & physical activity (MVPA)
